# Stakeholder views regarding ethical issues in the design and conduct of pragmatic trials: study protocol

**DOI:** 10.1186/s12910-018-0332-z

**Published:** 2018-11-20

**Authors:** Stuart G. Nicholls, Kelly Carroll, Jamie Brehaut, Charles Weijer, Spencer Phillips Hey, Cory E. Goldstein, Merrick Zwarenstein, Ian D. Graham, Joanne E. McKenzie, Lauralyn McIntyre, Vipul Jairath, Marion K. Campbell, Jeremy M. Grimshaw, Dean A. Fergusson, Monica Taljaard

**Affiliations:** 1 Clinical Epidemiology Program, Ottawa Hospital Research Institute, Civic Campus, 1053 Carling Avenue, Civic Box 693, Admin Services Building, ASB 2-013, Ottawa, ON K1Y 4E9 Canada; 2 Rotman Institute of Philosophy, Western University, London, ON Canada; 3 Center for Bioethics, Harvard Medical School, Boston, MA USA; 4 Program on Regulation, Therapeutics and Law (PORTAL), Brigham and Women’s Hospital, Boston, MA USA; 5 Rotman Institute of Philosophy, Western University, London, ON Canada; 6 Centre for Studies in Family Medicine, Department of Family Medicine, Schulich School of Medicine & Dentistry, Western University, London, ON Canada; 7 School of Public Health and Preventive Medicine, Monash University, Melbourne, Australia; 8 Department of Medicine, Division of Gastroenterology, Western University, London, ON Canada; 9 Department of Epidemiology and Biostatistics, Western University, London, ON Canada; 10 Health Services Research Unit, University of Aberdeen, Aberdeen, UK; 11 Department of Medicine, University of Ottawa, Ottawa, ON Canada; 12 Faculty of Medicine, University of Ottawa, 501 Smyth Road, Box 201B, Ottawa, ON K1H 8L6 Canada; 13 School of Epidemiology and Public Health, University of Ottawa, Ottawa, ON Canada

## Abstract

**Background:**

Randomized controlled trial (RCT) trial designs exist on an explanatory-pragmatic spectrum, depending on the degree to which a study aims to address a question of efficacy or effectiveness. As conceptualized by Schwartz and Lellouch in 1967, an explanatory approach to trial design emphasizes hypothesis testing about the mechanisms of action of treatments under ideal conditions (efficacy), whereas a pragmatic approach emphasizes testing effectiveness of two or more available treatments in real-world conditions. Interest in, and the number of, pragmatic trials has grown substantially in recent years, with increased recognition by funders and stakeholders worldwide of the need for credible evidence to inform clinical decision-making. This increase has been accompanied by the onset of learning healthcare systems, as well as an increasing focus on patient-oriented research. However, pragmatic trials have ethical challenges that have not yet been identified or adequately characterized. The present study aims to explore the views of key stakeholders with respect to ethical issues raised by the design and conduct of pragmatic trials. It is embedded within a large, four-year project that seeks to develop guidance for the ethical design and conduct of pragmatic trials. As a first step, this study will address important gaps in the current empirical literature with respect to identifying a comprehensive range of ethical issues arising from the design and conduct of pragmatic trials. By opening up a broad range of topics for consideration within our parallel ethical analysis, we will extend the current debate, which has largely emphasized issues of consent, to the range of ethical considerations that may flow from specific design choices.

**Methods:**

Semi-structured interviews with key stakeholders (e.g. trialists, methodologists, lay members of study teams, bioethicists, and research ethics committee members), across multiple jurisdictions, identified based on their known experience and/or expertise with pragmatic trials.

**Discussion:**

We expect that the study outputs will be of interest to a wide range of knowledge users including trialists, ethicists, research ethics committees, journal editors, regulators, healthcare policymakers, research funders and patient groups. All publications will adhere to the Tri-Agency Open Access Policy on Publications.

**Electronic supplementary material:**

The online version of this article (10.1186/s12910-018-0332-z) contains supplementary material, which is available to authorized users.

## Background

Providing the best available care to patients is the backbone of medical practice, and a practical consequence of the ethical principles of non-maleficence and beneficence [1, 2]. Ideally, medical care should be grounded in valid evidence of benefit, safety and cost-effectiveness [3]. A gold standard study design for providing evidence of effectiveness is the randomized controlled trial (RCT) [4].

Randomized controlled trials differ in their purpose and scope and accordingly, can have different methodological approaches. Trials that focus on elucidating a mechanism of action or efficacy under highly-controlled conditions are often described as explanatory or mechanistic trials (herein ‘explanatory RCTs’) [5–8]. Conversely, trials whose outcomes are measuring intervention effectiveness in a real-world setting, and where the aim is to provide information pertinent to a health care decision, have been described as “practical” [6, 9] or pragmatic (herein ‘pragmatic RCTs’) [5, 10–13]. In reality, individual trials will lie somewhere along the spectrum from more explanatory to more pragmatic depending on the degree to which their aim(s) and study design are more aligned to the study of efficacy or effectiveness.^1^ To help trialists match their design decisions to the purpose of the trial, tools such as the Pragmatic-Explanatory Continuum Indicatory Summary (PRECIS) and its update PRECIS-2 [14] have been developed. PRECIS-2 proposes nine discrete domains in which trialists can make design decisions for the design of pragmatic RCTs. These domains are summarized in Table 1. In general, the design of an RCT should be driven by the desired purpose [6, 9].

**Table 1:** PRECIS-2 domains and descriptors

PRECIS-2 domain	Description
Eligibility	To what extent are the participants in the trial similar to those who would receive this intervention if it was part of usual care? A more pragmatic trial would have criteria that ensure participants are essentially identical to those in usual care; a more explanatory approach would have lots of exclusions (e.g. those who don’t comply, respond to treatment, or are not at high risk for primary outcome, are children or elderly), or uses many selection tests not used in usual care.
Recruitment	How much extra effort is made to recruit participants over and above what that would be used in the usual care setting to engage with patients? For example, a very pragmatic trial may have recruitment through usual appointments or clinic; a very explanatory trial may have targeted invitation letters, advertising in newspapers, radio plus incentives and other routes that would not be used in usual care.
Setting	How different is the setting of the trial and the usual care setting? For example, a very pragmatic trial may use identical settings to usual care; a very explanatory trial may include a single centre, or only specialised trial or academic centres.
Organisation	How different are the resources, provider expertise and the organisation of care delivery in the intervention arm of the trial and those available in usual care? For example, a very pragmatic trial may use identical organisation to usual care; a very explanatory trial may increase staff levels, give additional training, require more than usual experience or certification and increase resources.
Flexibility (delivery)	How different is the flexibility in how the intervention is delivered and the flexibility likely in usual care? For example, a very pragmatic trial may have identical flexibility to usual care allowing healthcare professionals to modify delivery of the intervention; a very explanatory trial may include a strict protocol, monitoring and measures to improve compliance, with specific advice on allowed co-interventions and complications
Flexibility (adherence)	How different is the flexibility in how participants must adhere to the intervention and the flexibility likely in usual care? For example, a very pragmatic trial may involve no more than usual encouragement to adhere to the intervention; a very explanatory approach may involve exclusion based on adherence, and measures to improve adherence if found wanting.
Follow-up	How different is the intensity of measurement and follow-up of participants in the trial and the likely follow-up in usual care? For example, a very pragmatic trial may have no follow up than would be the case in usual care; a very explanatory approach may have more frequent, longer visits, unscheduled visits triggered by primary outcome event or intervening event, and more extensive data collection.
Primary outcome	To what extent is the trial’s primary outcome relevant to participants? For example, a very pragmatic trial would have an outcome is of obvious importance to participants; a very explanatory trial may use a surrogate, physiological outcome, central adjudication or use assessment expertise that is not available in usual care, or the outcome is measured at an earlier time than in usual care.
Primary analysis	To what extent are all data included in the analysis of the primary outcome? For example, a very pragmatic trial would use intention to treat with all available data; a very explanatory analysis may exclude ineligible post-randomisation participants, or include only completers or those following the treatment protocol

Adapted from Loudon K, Treweek S, Sullivan F, Donnan P, Thorpe KE, Zwarenstein M. The PRECIS-2 tool: designing trials that are fit for purpose. *BMJ*. 2015;350:h2147 and https://www.precis-2.org/Help/Documentation/HowTo

While the notion of more explanatory and more pragmatic attitudes within trials was first raised over 50 years ago [5], interest in pragmatic RCTs has grown substantially in recent years. Bibliometric analyses illustrate year on year increases in the number of publications with pragmatic trials as their topic [15, 16]. Further, a recent Delphi survey of 48 UK Clinical Trials Units (CTUs) found that over 40% of CTU Directors ranked pragmatic trials as a critical topic of interest [17]. Similarly, in a prioritization exercise among researchers and methodologists working on trials in low and middle income countries, 84% of respondents indicated that methodological research regarding pragmatic trials was a critical research topic [18].

There are several reasons for this increased interest. First, funding agencies such as the Patient Centered Outcomes Research Institute (PCORI) and the Canadian Institutes of Health Research (CIHR), among others, have moved toward funding more pragmatic research questions. Second, there may be a lack of evidence for established practices – a form of “self-evident evidence paradox” [19] – that is now coming under closer scrutiny. Third, there are now many new or emerging trial designs that capitalize on methodological and statistical innovations as well as the expansion in availability of routinely-collected health data. This may enhance opportunities for pragmatism in key domains of design such as identification and recruitment of participants, the intensity of follow up required, and the collection of outcome data. Examples of designs that may lend themselves to more pragmatic approaches include novel cluster randomized trials (such as cluster crossovers [20, 21] and stepped wedge trials), cohort multiple randomized design [22], and registry-based randomized controlled trials [23]. Fourth, the cost and logistical complexity of traditional explanatory trials has motivated funders and researchers to identify potential ways to reduce trial costs, with pragmatic RCTs that potentially leverage existing infrastructure – such as health administrative data – seen as a one way to do this [24–26]. Finally, selection of more patient-focused trial outcomes closely aligns to increasing interest in, and funding for, patient engagement and patient-oriented research with national strategies such as the Canadian Strategy for Patient Oriented Research (SPOR) [27] and the US Patient-Centered Outcomes Research Institute (PCORI) [28] (see Table 2 for examples).

**Table 2:** Statements regarding pragmatic trials by funding agencies

Agency	Year	Definition of pragmatic trial
Canadian Institutes of Health Research (Canada)	2016	"[innovative Clinical Trials (iCT)] methods can reduce the cost of conducting trials, reduce the amount of time needed to answer research questions, and increase the relevance of research findings to patients, health care providers and policy makers. Adopting these alternative designs can maximize the use of existing knowledge and data. Some examples of iCTs include: Pragmatic trials [...];" https://www.researchnet-recherchenet.ca/rnr16/vwOpprtntyDtls.do?prog=2471&view=search&terms=pragmatic&incArc=true&org=CIHR&fromYear=2005&toYear=2022&type=EXACT&resultCount=25&next=1
Medical Research Council (UK)	2018	“Effectiveness Trials - Studies designed to produce research information about the effectiveness, costs, and broader impact of health technologies for those who use, manage and provide care in the NHS are supported by the Health Technology Assessment (HTA) programme, funded by NIHR and managed by NETCC.” https://mrc.ukri.org/funding/science-areas/translation/translation-and-clinical-trials/
NCCIH (US)	2017	“The projects must be pragmatic trials rather than explanatory trials. […] The Collaboratory supports pragmatic trials. A pragmatic trial is primarily designed to determine the effects of an intervention under the usual, real-world conditions in which it will be applied. The approach, including study design, is kept as simple as possible without sacrificing scientific rigor.” https://nccih.nih.gov/news/events/telecon/pragmatic-CT-webinar-rm-16-019
PCORI (US)	2018	“[…] applicants should design trials so that they address practical comparative questions faced by patients and clinicians—to include broader and more diverse populations—and can be conducted in real-world clinical and diverse health-system settings. Such trials are often referred to as “pragmatic clinical trials” because they are intended to provide information that healthcare providers can adopt directly.” https://www.pcori.org/sites/default/files/PCORI-PFA-2018-Cycle-1-Pragmatic-Studies.pdf

### Ethical issues in pragmatic RCTs

Pragmatic trial designs may, however, also raise new ethical challenges. Consider the design domain of recruitment: a more pragmatic RCT may attempt to recruit participants by utilizing clinical staff during routine clinical encounters. This has the potential advantages of lowering trial cost while increasing expediency, but it also has the potential to blur the line between clinical practice and research and may raise concerns regarding the voluntariness of patient informed consent. On the other hand, a more explanatory trial with dedicated research staff responsible for recruiting patients is likely more costly, time consuming, and may result in greater recruitment challenges, but at the same time may provide a clearer separation between research and clinical care, and thus, more independence in the consent process. Further, recruitment undertaken by researchers may also provide more clarity about the ethical guidelines that should be adhered to in the research as opposed to a situation in which healthcare professionals recruit participants within routine clinical practice.

Other examples include the domain of eligibility criteria: Populations — such as pregnant women or older patients — are routinely excluded from clinical trials [9, 14, 29–31], and are thus exposed to risks in their clinical care brought about by a lack of evidence-informed practice. These populations would benefit from improved evidence gained by broader inclusion criteria within pragmatic RCTs. However, their inclusion may raise concerns as to whether additional protections need to be in place during the trial (such as closer monitoring) and if so, when, or how identification of potentially vulnerable populations should take place and what appropriate responses might be.

Finally, consider the domain of data collection: pragmatic trials commonly utilize routinely-collected data for outcome assessment. These systems were not designed for the purpose of capturing data for research purposes, and questions have been raised regarding the ability of electronic health record systems to comply with requirements set out within international research standards such as Good Clinical Practice [32–34].

There is now an emerging body of empirical research exploring these and other ethical challenges with more pragmatic RCTs [35–41]. Kalkman and colleagues [35], for example, identified four key themes through interviews with key stakeholders: that less controlled experimental conditions create safety concerns regarding the patients enrolled; that comparison between an intervention and a comparator that constitutes suboptimal usual care may compromise clinical equipoise; that consent processes may be modified, but the circumstances and extent of modification are contested; and that minimal interference with real-world practice drives arms to equivalence (and thus the trial may not find a statistically or clinically significant result).

In particular, questions of consent in pragmatic trials have been consistently raised as topics of interest and have been studied through vignette-based research with healthcare professionals and patients [36, 37], interviews with physicians [39], deliberative engagement activities [42] and surveys [41]. For example, in a study using case vignettes, respondents preferred specific disclosure and options to either opt-in or opt-out of research compared to a general policy in which patients were informed broadly that a healthcare system engages in Comparative Effectiveness Research (CER) [37, 42] . Similarly, a study examining the nature of CER as research or practice explored whether physicians have a duty to participate in quality improvement (QI) CER as well as whether patient consent is required for physician-targeted interventions [38]. Other studies have explored attitudes toward consent under different trial scenarios. This work found that attitudes toward consent differed not only by the type of intervention, but also due to different design elements such as randomization and the degree to which the trial design departed from routine clinical care [39].

### Limits of the existing literature

Despite this emerging body of evidence, the current literature has several limitations. First, few empirical studies have been grounded in the actual *experiences* of participants with the design or conduct of trials that are more pragmatic. Further, studies have tended to compartmentalize designs into either pragmatic or explanatory categories. Given that trials are generally considered to exist on a continuum between more explanatory and more pragmatic trials, it makes little sense to try and isolate ‘pragmatic’ RCTs and identify ‘their’ ethical issues. Instead, it would seem more valuable to identify ethical issues that may emerge from particular trial designs, domains or dimensions of trial pragmatism. Second, the literature is dominated by discussions set within the US healthcare context, resulting in appeals mainly to US regulations [43–45]. Third, few studies explicitly address governance of pragmatic trials or the perspectives of research ethics committees who review these trials. Fourth, studies have tended to focus either on broad concepts such as the Learning Healthcare System [46, 47] or on a very limited number of ethical issues. As such, debates have either tended to lack concrete application or have been overly narrow and deep. For example, there has been a great deal of focus on consent [42, 46, 48–50], and insufficient attention paid to the ethical issues that arise from decisions regarding the pragmatic or explanatory nature of individual trial design elements.

There is, we believe, a need for constructive ethical guidance relating to more pragmatic design choices within RCTs. Identifying key ethical considerations and the ways in which they are aligned with pragmatic design decisions is an essential first step on the path to developing guidance for researchers, research ethics committees and other key stakeholders in the design and conduct of more pragmatic RCTs. It is crucial that identification of such ethical issues is grounded in the experiences of those directly involved in the design, implementation, and evaluation of more pragmatic RCTs.

## Aim

The present study aims to explore the views of pragmatic trial experts and key stakeholders (for example: trialists, ethicists, methodologists, chairs of research ethics committees, health system leaders, quality improvement experts, and patient representatives on research study teams) to generate a thorough understanding of the types of ethical issues arising in the practice of pragmatic trials from a variety of perspectives. It is the first of five planned studies embedded within a large, four-year multi-aim project that seeks to, ultimately, develop ethical guidance for the design and conduct of pragmatic trials. The protocol for the full study is published elsewhere [51]. Within the present study we will:

1. Explore the experiences of key stakeholders regarding ethical issues that arise in the design and conduct of pragmatic RCTs;
2. Identify ethical issues that arise from taking more pragmatic (as opposed to explanatory) approaches to design elements;
3. Elicit perspectives regarding the appropriate ethical oversight of RCTs and how this may differ between trials that are more pragmatic or more explanatory.

The results from this study will be used to formulate a typology of ethical issues arising from more pragmatic trials, to be addressed in the conceptual work within the larger project. It will also inform the development of data extraction items for a planned review of published pragmatic trials, questionnaire items for a planned survey with trialists and research ethics committees, and discussion items for focus group discussions with trial participants.

## Methods/design

The methods will involve semi-structured interviews with key stakeholders with experience or exposure to trials reflecting more pragmatic designs. Interviewees will be identified using a purposive sampling strategy, augmented through snowball sampling [52, 53].

### Participants

Participants will reflect a broad range of stakeholders in the design and conduct of clinical trials generally, and pragmatic RCTs in particular. Specifically, we will recruit participants within two broad groups of stakeholders: trial experts and lay members of study teams.

#### Trial experts

Trial experts will include trialists, ethicists, methodologists, chairs of research ethics committees, health system leaders, and quality improvement experts with experience of pragmatic trials. To be considered eligible, potential interviewees must be recognized experts in trials that are more pragmatic (e.g., have been an investigator on multiple RCTs considered to be pragmatic in design, have published papers addressing the ethical challenges in more pragmatic RCTs, have been engaged in work regarding the methodological development of more pragmatic approaches to RCTs or major trial designs, or have been engaged in the governance or oversight of RCTs considered to be more pragmatic in nature). Potential interviewees will be selected across a broad range of jurisdictions and clinical areas to reflect a range of experiences.

#### Lay members of study teams

Lay members of study teams (i.e., members of study teams who have lived experience of the condition under study or who represent the groups involved in the study or broader community at large) will be eligible if they have played a role in the development or implementation of a specific trial deemed to be more pragmatic in design or have been engaged in larger study teams to enhance the design, conduct, or implementation of trials that are more pragmatic in design.

The increasing inclusion of lay members in study teams of pragmatic trials reflects the underlying premise that trials that are more pragmatic should address outcomes and study questions relevant to patients and clinical practice. While previous studies of ethical issues raised by pragmatic trials have included patients and members of the public [37, 42], we are deliberately targeting lay members of study teams who are more likely to have been exposed to a pragmatic RCT and to have given consideration to implications of study design choices. Furthermore, the inclusion of lay members of study teams will improve the saliency of the study question and the breadth of experience upon which the participant could draw.

### Identification and recruitment

#### Trial experts

An initial sample of trial experts will be identified through our extensive investigator networks, publications, and association with known centers conducting trials that may be more pragmatic in nature. Potential participants will be selected to ensure a breadth of experience and representation from across the identified range of stakeholder groups. We will also ensure representation from Canada, the US, UK, France, Australia and Low and Middle Income Countries. We chose these jurisdictions because they represent countries in which the vast majority of pragmatic trials are conducted, they have a rich history in methodological developmental of pragmatic trial designs, and our team members have connections with experts and research ethics organizations in these countries which will help facilitate participation. Depending on the emerging themes, we may purposively sample additional stakeholders to ensure a diversity of opinion is sought.

Initial contact (and subsequent follow up if necessary) with trial experts will be made via email by the study team. The initial contact will introduce the study design and purpose and inquire about the potential informant’s willingness to participate. If the potential interviewee is willing to participate, the interviewer (SN, KC) will arrange a time for the interview. Following confirmation of an interview date, an overview of the interview structure will be sent to participants together with a summary of the PRECIS-2 domains. On the agreed date, the consent form will be reviewed, and verbal consent will be obtained to proceed with the interview.

#### Lay members

To begin with, lay members of study teams will be identified via existing funded pragmatic trials or studies addressing the design, conduct or implementation of pragmatic trials which include lay members as part of the study team. Examples include trials which, due to funding requirements, require patient engagement (e.g. Ontario SPOR Support Unit Impact Awards, Patient-Centered Outcomes Research Institute (PCORI)) or through existing lists (e.g. PRECIS-2 list of evaluated trials: https://www.precis-2.org/Trials), or from investigator networks. This initial list will be supplemented through snowball sampling.

**Fig. 1:**
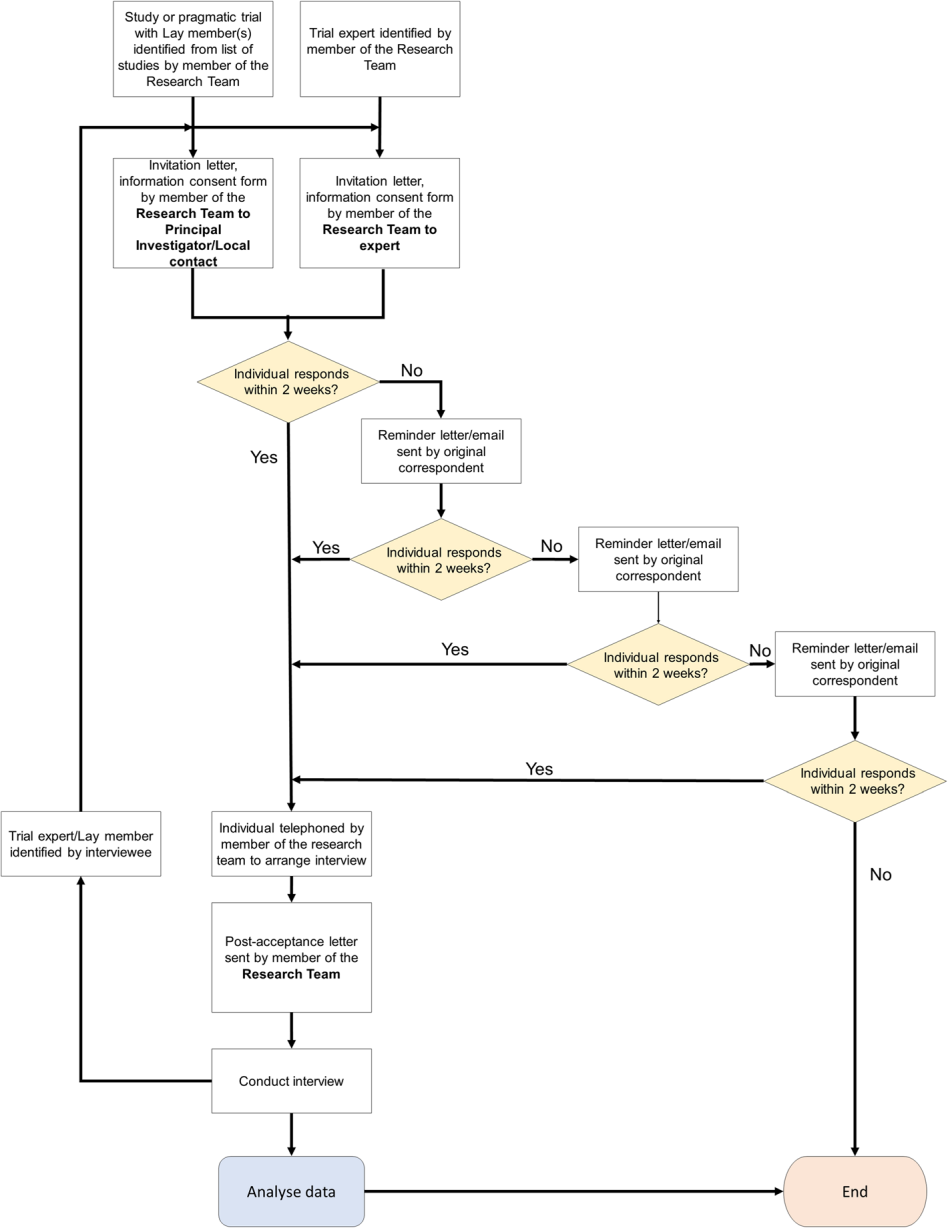
Interview recruitment process

The principal investigators for the identified studies will be approached via email and asked to either provide the contact information of the lay members involved in their trial, or to forward a study invitation and consent form on behalf of our investigator team. If the lay member of the study team is willing to participate, the interviewer (SN, KC) will arrange a time for the telephone interview. On the agreed date, the consent form will be reviewed, and verbal consent will be obtained to proceed with the interview. An overview of the recruitment process is presented in Fig. 1.

In all cases, potential interviewees will receive a copy of the consent form which will include an email address and phone number that potential interviewees can use to contact the study team if they are interested in participating. All participants will be offered an honorarium ($100 CAD) in recognition of their participation.

### Interview guide and data collection processes

Data will be collected through semi-structured interviews. Interviews with stakeholders will comprise three main sections: (i) experiences with pragmatic trials, including experiences of ethical issues; (ii) perceptions of ethical issues relevant to pragmatic trials; and (iii) perspectives on oversight and regulation. Interview guides were developed based on a review of the literature as well as existing tools, such as PRECIS-2. Draft interview guides were prepared by the team and pilot tested on three pragmatic trial experts. Given the differing populations, separate interview guides were generated for the trial experts and lay members of study teams (See Additional files 1 and 2 for interview schedules).

Interviews will be conducted by one of two members of the team (SN/KC). Both interviewers will have experience conducting qualitative interviews, and pilot interviews were conducted with both team members present to ensure familiarity with the interview guide and consistency of approach. The interviewers will give prompts and ask clarifying questions in addition to the questions in the interview protocol. Interviews will be conducted either in person or over the telephone as required, depending on participant availability and logistics. We anticipate the interview sessions to take approximately 1 h.

In all cases, interviews will be audio-recorded with consent, transcribed verbatim by a professional transcription service, and imported into qualitative data analysis software (QSR International’s NVivo 10 qualitative data analysis Software [54]) for analysis. If a participant wishes to take part but does not wish the interview to be audio-recorded, then written notes will be taken. During the process of transcription, data will be de-identified and interview transcripts will be assigned a unique participant ID. Copies of transcribed interviews will be made available to interviewees for additional comments.

### Sample size

Qualitative approaches necessarily require small samples due to the complex nature of the data generated and the costs incurred by collection and analysis of the data [52, 55]. Following established qualitative research methods, our target sample size is estimated at what will achieve saturation (i.e., when new interviews cease to provide fresh information) [52, 56–58]. While approximated, sample sizes are based on the experience of the team [59, 60]. We anticipate that 12–20 interviews per group of stakeholders (trial experts and lay members of study teams) will be required before data saturation is reached [56], and hence a total of *n* = 24–40 interviews. However, as saturation of topics is the stated end-point, additional interviews may be required (and will be undertaken as necessary).

### Analysis

The examination of the transcripts will follow a thematic analysis approach [61, 62]. Under this methodology, textual data contained within transcripts are coded and labeled in an inductive manner. Using the constant comparison technique, data analysis occurs in parallel to the conduct of interviews, allowing for the interview guide to evolve and integrate emergent themes into future interviews and for greater exploration of these issues. Comparisons will be made within and across interviews allowing for the revision, combination or separation of codes in light of new data [63–65]. After an initial phase of open coding, individual codes will be grouped into overarching themes or constructs through a process of data reduction. Analysis will be facilitated by qualitative data analysis software (QSR International’s NVivo 10 qualitative data analysis Software [54]) to assist with the collation and management of codes and themes.

Specifically, the analyses will consider: how pragmatic RCTs are conceptualized and aspects identified as being definitive components within trials that are more pragmatic in design; ethical considerations in the design of trials that are more pragmatic in nature; aspects of trial design that generate ethical discussion, and; considerations in the oversight or regulation of trials that are more pragmatic in nature.

Interviews will be coded independently by two researchers (SN, KC) who will then discuss between themselves, before presenting their analyses to the broader team for comments and further discussion. This process of dual coding has been suggested as a qualitative comparator to traditionally quantitative notions of inter-rater reliability [66].

## Discussion

This study will address important gaps in the current empirical literature by identifying a comprehensive range of ethical issues pertaining to pragmatic RCTs. Interviews will be conducted with a broad range of stakeholders including trialists, ethicists, methodologists, chairs of research ethics committees, health system leaders, and lay members of study teams. The interview guide was designed to be grounded in the experiences of participants who are actively engaged in the design, conduct, or evaluation or more pragmatic RCTs, which will help ensure that the results are applicable to clinical research and practice, as opposed to being based on hypothetical scenarios. Furthermore, by opening up a broad range of topics for consideration within our parallel ethical analysis, we will extend the current debate beyond consent to the range of ethical considerations that may flow from specific design choices. As such, the larger program of work which this exploratory study informs [51] promises to provide practical advice and guidance to a range of stakeholders in the design and conduct of RCTs including researchers, research ethics committee members, and regulators.

A main operational issue in the present study will be the identification and recruitment of lay members of study teams who have had exposure to pragmatic RCTs, and thus experience upon which to draw within the interview. To this end we have established connections with other stakeholder groups and identified funded trials that are pragmatic in nature and which, due to funding requirements, require patient engagement. This will ensure that all lay members of study teams that are approached will have experience of an RCT that is more pragmatic in design.

We thus expect that the study outputs will be of interest to a wide range of knowledge users including trialists, ethicists, research ethics chairs, journal editors, regulators, healthcare policymakers, research funders and patient groups.

## Additional files

Additional file 1: Trial expert interview guide. (DOC 87 kb)

Additional file 2: Lay member of study team interview guide. (DOC 74 kb)

## Abbreviations

CIHR: Canadian Institutes of Health Research
PCORI: Patient Centered Outcomes Research Institute
RCT: Randomized controlled trial
REC: Research ethics committee
SPOR: Strategy for Patient-Oriented Research

## Footnotes

1 While we acknowledge that trials that exist on a spectrum, for simplicity we use ‘explanatory RCTs’ to refer to those RCTs that are more explanatory and ‘pragmatic RCTs’ to refer to those that are more pragmatic.
