# Supplementary material for: Stakeholder views regarding ethical issues in the design and conduct of pragmatic trials: study protocol
Source: BMC Med Ethics. 2018 Nov 20;19:90. doi: 10.1186/s12910-018-0332-z (PMC6247737; doi:10.1186/s12910-018-0332-z)
Supplement: Supplementary file 1 — Trial expert interview guide. (DOC 87 kb) [file 12910_2018_332_MOESM1_ESM.doc]

Additional file 1: interview schedule – Trial Experts

Note: *Italicised* text is standard text for the interviewer.

Normal text is question wording or examples of probes

**BOLD/ CAPITALISED** text is example of where study specific text will be added

Red Text indicates relevant to preliminary framework or PRECIS-2 domains.

**[INTERVIEWER NOTE: REMEMBER: Focus is on identifying ethical PROBLEMS not SOLUTIONS]**

*Thank you for agreeing to be interviewed today. As discussed, and detailed in the consent form, our study is seeking to explore what key stakeholders see as important ethical issues in the design and conduct of pragmatic trials.*

*As was outlined in the letter confirming the interview date, what I would like to do today is explore your experiences regarding trials that are pragmatic in nature, what you feel are the pertinent ethical issues that these particular types of trials raise, and what you feel are the relevant issues relating to the ethics oversight of these sorts of trials.*

*You should also have received a copy of the PRECIS-2 diagram that includes an explanation of specific domains on which a trialist can make design choices to push a trial to be either more pragmatic, or more explanatory. We will refer to these domains as a way into discussion of ethical issues raised by specific design aspects of more pragmatic trials.*

*Before I start, do you have any questions about the study or the interview?*

[**Address any questions**]

*OK, thank you.*

**[Get verbal agreement to proceed; including consent to audio recording]**

*Thank you.*

**PART 1: INTRODUCTION – EXPLORING EXPERIENCE(S) AND DEFINING SCOPE (10 minutes)**

Q. I was wondering if you could start by telling me a bit about your experience with trials that are more pragmatic in nature. So what can you tell me about the types of pragmatic trials you have been involved with?

**PROBE: e.g.** For example, have they been mostly trials of patient therapeutic interventions, or health policy and systems interventions?

Q. In what capacity (or capacities) have you been involved in these pragmatic trials?

Q. How many pragmatic trials (approximately) have you been involved with and over what number of years?

Q. Have these involved pragmatic trials in Low and Middle Income Countries?

Q. When you think of a trial that is more pragmatic, what comes to mind?

**PROBE: e.g**. What is your prototype of a ‘pragmatic’ trial?

**Part II: DESIGN, CONDUCT, AND ETHICAL IMPLICATIONS (40-60 minutes)**

Q. Can you tell me about the typical level of discussion about **ethical issues** in the design and/or conduct of your pragmatic trials?

Q. Would you say there are particular design aspects of more pragmatic trials that tend to generate discussion of ethical issues?

**ELEMENTS OF PRAGMATISM**

*A tool that has been developed to help researchers and trialists think through design decisions is the PRECIS -2 tool which was sent to you in the introductory email. Drawing on this tool, I would like to get your thoughts on what ethical issues might arise from taking a more pragmatic approach to trial design.*

**[PROBE ONLY ASPECTS NOT ALREADY SPONTANEOUSLY RAISED]**

| **PRECIS-2 Domain** | **Question** |
| --- | --- |
| **Eligibility** | *An aspect that has been identified as making a trial more pragmatic is the broadening of eligibility criteria with the aim of recruiting patients that better reflect those who would receive the intervention in usual care.*  Q. When do you tend to see ethical concerns raised by choices of inclusion criteria?  **PROBE**: For example, does a trial with broader inclusion criteria raise any ethical advantages or concerns?  Q. Can you think of any specific examples where broader inclusion criteria have been a topic of discussion from an ethics perspective?  Q. How might the ethical issues differ between trials that are more pragmatic versus more explanatory?  **PROBE**: Do you tend to see such concerns raised more or less frequently in trials with broader inclusion criteria? |
| **Recruitment** | *Trials that are more pragmatic can also differ from more explanatory trials with respect to the effort needed for recruitment. In more pragmatic trials, for example, patients might be recruited during scheduled clinic visits. This is in contrast with more explanatory trials where recruitment efforts are more extensive, possibly including advertising, incentives, or detailed searches of medical record for eligible patients.*  Q. When do you tend to see ethical concerns generated by the recruitment process?  **PROBE**: For example, if patients are recruited during a routine clinic visit, would you see any differences from an ethics perspective, to, say, a written invitation inviting them to the study?  Q. Can you think of any specific examples where a more pragmatic approach to recruitment has been a topic of discussion from an ethics perspective?  Q. How might the ethical issues around recruitment differ between more pragmatic trials compared with more explanatory trials?  **PROBE:** Do you tend to see concerns raised more or less frequently in trials that are more pragmatic in the domain of recruitment? |
| **Setting & Organisation** | *Several parts of the PRECIS-2 diagram refer to the delivery of the intervention. More explanatory trials may have dedicated study staff to undertake study procedures, which may take place in specialist study site while in more pragmatic trials no dedicated study staff may be provided and the study may take place in the usual care setting.*  Q. What ethical considerations might the study setting or organisation raise?  **PROBE:** For example, do you see any ethical issues raised by a study which uses specially trained study staff delivering the intervention compared to clinical care staff?  Q. Can you think of any specific examples where more pragmatic approaches to the setting or organisation of the study have been a topic of discussion from an ethics perspective?  PROBE: Does an LMIC context raise any relevant or additional issues with respect to this domain?  Q. How might the ethical issues regarding the setting or organisation of care differ between more pragmatic trials compared with more explanatory trials?  **PROBE:** Do you tend to see concerns raised more or less frequently in trials that are more pragmatic in the domain of setting or organisation? |
| **Intervention/Comparator**  **(GETREAL)** | *A key way that more pragmatic trials can differ from more explanatory trials is the choice of the intervention or comparator. In trials with more pragmatic aims, the intervention or comparator arms may be defined in terms of usual care, while in trials with more explanatory aims, the intervention arm may be experimental and the comparator arm may be placebo.*  Q. What would you say are important ethical considerations when deciding on the choice of the intervention or comparator?  **PROBE:** For example, do you see any particular ethical issues arising from the choice of usual care as the intervention or comparator?  Q. Can you think of any specific examples where the choice of the intervention or comparator was a topic of discussion from an ethics perspective?  Q. How might the ethical issues regarding the intervention or comparator differ between more pragmatic trials compared with more explanatory trials?  **PROBE:** Do you tend to see concerns raised more or less frequently in trials that are more pragmatic in the domain of intervention/comparator?  *Usual care may be defined in terms of frequency of use in clinical practice; reference to guidelines or best practice; or by individual practice.*  Q. Do you see any ethically important differences between these definitions? |
| **Flexibility (delivery)**  **Flexibility (adherence) (PRECIS-2)**  **Participant (GETREAL)** | *Another aspect identified as making trials more or less pragmatic is the level of autonomy in providing the intervention. In more pragmatic trials the intervention or control arm is delivered with greater flexibility compared to a more protocolised study in which delivery is tightly controlled. Similarly, in more pragmatic trials there is flexibility in adherence, with few if any special measures to ensure engagement or compliance with the intervention. This is in contrast to a more explanatory trial where procedures are in place to ensure or promote compliance (and which go beyond those used in usual care).*  Q. When do you tend to see any ethical issues arising from the way in which the intervention is delivered?  **PROBE:** For example, if a trial allows a clinician to prescribe the intervention flexibly using their own clinical judgement and there is little effort to ensure full compliance, does it raise any ethical concerns compared to a trial that requires the intervention to be delivered in a protocolised way and takes steps to ensure compliance?  Q. Can you think of any specific examples where a more pragmatic approach to the delivery of the intervention (or comparator) has been a topic of discussion from an ethics perspective?  Q. How might the ethical issues differ between more pragmatic trials and more explanatory trials in terms of the delivery of the intervention (or comparator)?  **PROBE:** Do you tend to see concerns raised more or less frequently in trials that are more pragmatic in the domains of delivery or adherence? |
| **Follow up** | *In more pragmatic trials the intensity of follow-up for participants may be no greater than that of usual care. This is in contrast to more explanatory trials where there may be extensive follow up of participants, for example through additional visits or more extensive data collection.*  Q. When do you see any ethical considerations arising from the approach to the follow up of participants?  **PROBE:** For example, does a more pragmatic trial which conducts follow up and collects outcomes from sources of routinely collected data, such as administrative databases or registries raise any ethical advantages or concerns?  Q. Can you think of any specific examples where a more pragmatic approach to follow up has raised a discussion of ethical issues?  Q. How might the ethical issues differ between more pragmatic and more explanatory trials in terms of the follow up of participants?  **PROBE:** Do you tend to see concerns raised more or less frequently in trials that are more pragmatic in the domains of follow up of participants? |
| **Primary Outcome** | *In a highly pragmatic trial the primary outcome might be one that is very relevant to participants such as quality of life or mortality; a very explanatory approach on the other hand might use a surrogate outcome or a physiological outcome that might not be as directly relevant to the participants.*  Q. What might you see as important ethical issues arising from the choice of primary outcome?  **PROBE:** For example, a more pragmatic trial may have quality of life or mortality as its primary outcome while an explanatory trial might have a level of a biomarker as its primary outcome.  Q. Can you think of any specific examples where the choice of primary outcome was a topic of discussion from an ethics perspective?  Q. How might the ethical issues differ between more pragmatic trials and more explanatory trials with respect to the choice of primary outcome? |
| **Analysis** | *In a trial with more pragmatic aims, all participants who entered the trial are analysed according to their randomized group (called analysis by “intent to treat”). In contrast, a trial with more explanatory aims might try to justify excluding participants who did not complete the trial or did not adhere to the protocol.*  Q. What would you say were the relevant ethical considerations relating to the analytic approach adopted?  **PROBE**: For example, would you have more or fewer concerns about a strict analytical approach involving all participants according to their allocated group?  Q. Can you think of any specific examples where the analytic approach has been a topic of discussion from an ethics perspective?  Q. How might the ethical issues differ between more pragmatic trials and more explanatory trials with respect to the analytic approach? |

Q. Looking at the PRECIS-2 diagram, would you say there are any elements that are missing and which are important in differentiating more explanatory trials from more pragmatic trials?

Q. Would you say that differences in **[RESPONSE]** raise any unique ethical issues in more pragmatic trials?

**PROBE: e.g.** What is it about [**RESPONSE**] that creates this ethical issue? What difficulties would you see a more pragmatic trial creating with respect to that particular domain?

Q. Thank you. We’ve talked about a number of specific design choices relevant to how pragmatic a trial is and the ethical consequences of these design choices. However, a fundamental decision that needs to be made as a first step is whether a pragmatic or explanatory trial is needed to answer the research question. So I would like to ask, do you see any particular contexts or circumstances that legitimise the choice to conduct a more pragmatic versus more explanatory trial, or conversely, that preclude conducting a more pragmatic versus more explanatory trial?

**PROBE:** For example, do you think there are ethical principles that support the choice to conduct a more pragmatic trial that perhaps are not as relevant to more explanatory trials, or a certain level of evidence that is needed before a pragmatic trial should be conducted?

**PART III: OVERSIGHT AND REGULATION (10-20 minutes)**

*Thank you. For the last part of the interview I would like to change gears and talk about oversight and regulation of trials if I may.*

Q. Based on your experience, what would you say are the core ethical issues that arise in a more pragmatic trials that would be relevant to a research ethics committee’s decision-making?

Q. Do you think that there are any aspects of more pragmatic trials that receive undue consideration from research ethics committees?

Q. Do you think that there are any aspects of more pragmatic trials that do not receive enough consideration from research ethics committees?

**CONCLUSION AND SNOWBALLING**

Q. Thank you, that’s all I wanted to ask you today. During our discussion did anything else come to mind that we haven’t talked about, or is there anything you would like to revisit?

Q. Thank you. One final question I have is about where we might find other perspectives on pragmatic trials. Would you have any recommendations about other people we could talk to, and who may provide other or different perspectives on the ethical issues of pragmatic trials? This can be in relation to design, ethical practices, or even the views from lay or public members of study teams.

***[If any names provided thank the participant and ask if you can mention their name when you follow up with the identified person.]***
