# Supplementary material for: Stakeholder views regarding ethical issues in the design and conduct of pragmatic trials: study protocol
Source: BMC Med Ethics. 2018 Nov 20;19:90. doi: 10.1186/s12910-018-0332-z (PMC6247737; doi:10.1186/s12910-018-0332-z)
Supplement: Supplementary file 2 — Lay member of study team interview guide. (DOC 74 kb) [file 12910_2018_332_MOESM2_ESM.doc]

**Additional file 2: interview schedule – Lay members of study teams [generic]**

Note: *Italicised* text is standard text for the interviewer.

Normal text is question wording or examples of probes

**BOLD/ CAPITALISED** text is example of where study specific text will be added

Red Text indicates relevant to preliminary framework or PRECIS-2 domains.

**[INTERVIEWER NOTE: REMEMBER: Focus is on identifying ethical PROBLEMS not SOLUTIONS]**

*Thank you for agreeing to be interviewed today. As discussed, and as you will have seen in the consent form, we are looking to talk to people who have been involved in clinical research, and specifically what issues arise when designing or conducting research.*

*In particular, we wanted to speak with you because of your experience with the* ***[STUDY]****. What I would like to do today is explore your experience of that particular trial, the issues that might have come up during the trial, and how the study team dealt with these. I would then like to discuss some other issues that have been raised with respect to trials like the* ***[STUDY]*** *and get your thoughts on these. Does that sound OK?*

*Before I start, do you have any questions about the study or the interview?*

**[Address any questions]**

*OK, thank you.*

**[Get verbal agreement to proceed; including consent to audio recording]**

*Thank you.*

**PART I: EXPLORING EXPERIENCES (10 minutes)**

Q. Thank you. I understand that you have been involved in the [**STUDY**]. Can you start by telling me how you got involved in [**STUDY**]

*Lay members of study teams – like researchers - can be involved in a study at different stages, and with involvement in some aspects more than others. This can range from defining or refining the research question all the way through to interpretation of data analysis and writing up the study.*

Q. Can you start by telling me what your role was in the [**STUDY**] and how you were involved at the different stages?

***[CHECK OFF THE FOLLOWING SECTIONS : [Based on listed stages of research engagement by INVOLVE UK] :***

***- Defining/refining the research question? - Planning/developing the grant proposal?***

***- Participating in discussion of study design, outcome measure selection?***

***- The REB application? - Data collection, analysis/interpretation?***

***- Dissemination?***

Q. Based on your experience with [**STUDY**] was there generally much discussion of **ethical issues or concerns** during your involvement?

**Q. IF YES**: What sort of issues came up?

**Part II: DESIGN, CONDUCT, AND ETHICAL IMPLICATIONS (40 minutes)**

*Thank you. We have talked a bit about your experience with [****STUDY].*** *I would now like to talk through some of the different aspects of [****STUDY****], and get your thoughts on these, and whether you see different choices that researchers make during the design of the study as raising ethical concerns.*

ELEMENTS OF PRAGMATISM

| **Domain** | **If applicable to study in question** | | **Alternate (if not applicable)** |
| --- | --- | --- | --- |
| **Eligibility** | *Trials can differ in who can take part in the trial; some trials are more open with who can be part of the trial, others are more strict. For example, some trials might deliberately exclude sicker patients or pregnant women. The rules about who gets to be part of the trial are called the eligibility criteria.* | | |
|  | Q. In the [**STUDY**] **[ELIGIBILITY CRITERIA]**; that is, the rules about who could be included, were quite broad. In other studies, these rules might be very specific so that only selected patients are included.  Q. Do you recall if there was much discussion about who would be eligible to take part in the study?  **IF YES:** Q. Were particular concerns raised about this? What were your thoughts? | | Q. In the [**STUDY**] the **[ELIGIBILITY CRITERIA]**; that is, the rules about who could be included, were quite specific. Some studies have tried to recruit a broader set of patients that might better reflect the patients that would likely get the intervention in practice.  Q. Do you recall if there was much discussion about who would be eligible to take part in the study?  **IF YES:** Q. Were particular concerns raised about this? What were your thoughts? |
| **Recruitment, Setting, Organisation** | *Clinical trials can also differ in how people are recruited into the trial. For example, patients might be recruited by healthcare professionals during a normal clinic visit. This is different to other trials where recruitment might include advertising, financial incentives, or the use of trained specialist staff to find and recruit eligible patients.* | | |
|  | Q . The [**STUDY**] used **[RECRUITMENT DETAILS]** instead of independent research staff.  Q. Do you recall if there was much discussion about how recruitment would happen?  **IF YES:** Q. Were particular concerns raised about this? What were your thoughts? | | Q . The [**STUDY**] used [**RECRUITMENT DETAILS**] to enroll patients into the study. In some studies, patients might be recruited during a routine clinic visit, or by healthcare professionals without specific research study staff.  Q. Do you recall if there was much discussion about how recruitment would happen?  **IF YES:** Q. Were particular concerns raised about this? What were your thoughts? |
| **Intervention/Comparator**  **(GETREAL)** | *In some studies the trial might compare treatments (for example, drugs) that are already being widely used. In other studies the trial might test a new (unproven) treatment against the best available treatment already being used, or against something else. These treatments that are already being widely used are called “usual care” treatments.* | | |
|  | Q. In the **[STUDY**] the [**STUDY INTERVENTIONS**] were [**DESCRIBE INTERVENTION AND COMPARATOR**].  Q. Do you recall there being much discussion about the choice of the treatment options or about ‘usual care’?  **IF YES: Q**. Were particular concerns raised about this? What were your thoughts? | Q. In the **[STUDY**] the [**STUDY INTERVENTIONS**] were [**DESCRIBE INTERVENTION AND COMPARATOR**].  Q. Do you recall there being much discussion about the choice of the treatment options or about ‘usual care’?  **IF YES: Q**. Were particular concerns raised about this? What were your thoughts? | |
| **Flexibility** | *In some studies the doctors or healthcare professionals participating in the trial have very specific instructions about how the treatments must be used, and all patients included in the trial must get the treatment in the exact same way. Doctors might also be closely monitored by the researchers to make sure that the treatments are being used as they are supposed to. In other studies the doctors have flexibility and can adapt and alter how they deliver the treatments.* | | |
|  | Q. In the **[STUDY**] the [**STUDY INTERVENTIONS**] were [**DESCRIBE ASSIGNMENT**].  Q. Do you recall there being much discussion about how participants would be given the intervention or whether there would be monitoring to see if the intervention was used as it was supposed to be?  **IF YES: Q**. Were particular concerns raised about this? What were your thoughts? | | Q. In the **[STUDY**] the [**STUDY INTERVENTIONS**] were used in a specific way determined by a protocol. All patients got the intervention in the exact same way. In other studies, there is more flexibility and the patient’s doctor can have flexibility to give or not give the treatment as they see fit.  Q. Do you recall there being much discussion about how participants would be given the intervention or whether there would be monitoring to see if the intervention was used as it was supposed to be?  **IF YES: Q**. Were particular concerns raised about this? What were your thoughts? |
| **Follow up** | *In some trials the follow-up of participants is no more than it would be in usual care, and all the trial results might come from information already being collected by the hospital as part of routine care. In other trials there might be a lot of extra follow- up with participants, for example, extra visits, or even surveys and extra tests.* | | |
|  | Q. In the [**STUDY**] the results came from data **[OUTCOMES**] routinely collected from the **[DATA SOURCE],** rather than from questionnaires completed by patients specifically for the research.  Q. Do you recall there being much discussion about researchers having access to this sort of data?  **IF YES: Q**. Were particular concerns raised about this? What were your thoughts? | | Q. In the [**STUDY**] the results came from data **[OUTCOMES**] specifically collected for the research study and not routinely collected. However, in some studies, the results are derived from data that is collected by healthcare professionals and recorded as part of routine care.  Q. Do you recall there being much discussion about researchers having access to this sort of data?  **IF YES: Q**. Were particular concerns raised about this? What were your thoughts? |
| **Primary outcome** | *In some trials the main outcome that is assessed is selected because it is would be very relevant to patients and healthcare professionals, for example quality of life or mortality. In other trials the main outcome measure might not be as directly relevant to the patients, for example the levels on a blood test.* | | |
|  | Q. In the [**STUDY**] the main outcome was [**PRIMARY OUTCOME**]  Q. Do you recall there being much discussion about what the main outcome should be?  **IF YES: Q**. Were particular concerns raised about this? What were your thoughts? | | Q. In the [**STUDY**] the main outcome was [**PRIMARY OUTCOME**]  Q. Do you recall there being much discussion about what the main outcome should be?  **IF YES: Q**. Were particular concerns raised about this? What were your thoughts? |

**PART III: OVERSIGHT AND REGULATION (10 minutes)**

**FRAMEWORK ISSUE: What research ethics oversight is required for pRCTs?**

*Thank you. For the last part of the interview I would like to change gears and talk about oversight and regulation of clinical trials if I may. Studies like the* ***[STUDY]*** *will have to go through an ethics review where the study is reviewed by a research ethics committee made up of other researchers (as well as people trained in law, ethics and members of the community) to identify any concerns with the design or the way the study is proposed to go ahead.*

**[SEE RESPONSES TO SECTION I. DO NOT ASK IF NOT RELEVANT]**

Q. As part of your role in **[STUDY]** were you involved in the ethics review or did you get to see the reviews from the research ethics committee?

**IF YES, PROBE**: Can you tell me a little about these encounters – were particular issues raised by the ethics review? How were these dealt with?

Q. Based on your experience of **[STUDY]**, what do you think are the main things that a research ethics committee should be looking at when they review research like **[STUDY**]?

**PROBE**: Why do you feel these are important? What is it about these aspects that warrant particular consideration?

**CONCLUSION AND SNOWBALLING**

Q. Thank you. I would like to ask you if there is anything we haven’t discussed and that you feel are relevant to **[STUDY]** or anything you would like to revisit? This might be an issue that was discussed by the research team, or one you think is relevant but hasn’t been raised.

Q. Thank you. One final question I have is about where we might find other perspectives on studies like the **[STUDY]?** Would you have any recommendations about other people we could talk to?

***[If any names provided thank the participant and ask if you can mention their name when you follow up with the identified person. ]***
